# Physical Activity, Cognitive Function, and Learning Processes: The Role of Environmental Context

**DOI:** 10.3390/bs15121630

**Published:** 2025-11-27

**Authors:** Francesca Latino, Giovanni Tafuri, Giulia Amato, Generoso Romano

**Affiliations:** 1 Department of Education and Sport Sciences, Pegaso University, 80143 Naples, Italy; 2 Department of Medical, Motor and Wellness Sciences, University of Naples “Parthenope”, 80133 Napoli, Italy

**Keywords:** physical activity, cognitive performance, stress levels, environmental stressors, outdoor

## Abstract

A growing body of evidence highlights the beneficial role of physical activity in supporting cognitive functions and learning outcomes. Yet, recent studies indicate that these effects may be shaped by environmental conditions, conceptualized within the framework of the urban exposome. The present study explores the interaction between physical activity, cognitive enhancement, and environmental exposures such as air pollution, noise, sensory overstimulation, and access to green spaces. A multi-method experimental design was implemented with 60 participants randomly assigned to either an experimental or a control group. The experimental group engaged in moderate-intensity physical activity across diverse urban settings, including green parks, high-traffic streets, and indoor facilities, while the control group performed the same activity in a stable indoor environment without environmental variability. Cognitive performance was assessed before and after physical activity through standardized measures of attention, memory, and executive function. Psychological and physiological stress responses were also monitored using the Perceived Stress Scale (PSS) and heart rate variability (HRV). Results suggest that the cognitive benefits of physical activity are not exclusively attributable to internal physiological mechanisms but are significantly moderated by environmental exposures. These findings underscore the relevance of considering contextual factors when examining the links between physical activity, cognition, and academic performance.

## 1. Introduction

In recent years, the relationship between physical activity and cognitive function has been the subject of increasing scientific attention (Álvarez-Bueno et al., 2017; Bidzan-Bluma & Lipowska, 2018; Hakun et al., 2025; Kekäläinen et al., 2023; Sewell et al., 2021). Physical activity has long been known to have numerous health benefits, including improving cardiovascular health, enhancing muscular strength, and boosting immune system function (Dhuli et al., 2022; Hallal et al., 2006; Nurzynska et al., 2013; Tafuri & Latino, 2024; Warburton & Bredin, 2017). However, its effects on cognitive performance, particularly in terms of learning, memory, and executive function, are now recognized as equally important (de Greeff et al., 2018; Latino et al., 2021; Singh et al., 2025; Tian et al., 2023). Physical activity has been shown to enhance brain plasticity, improve mood, and reduce stress, factors which collectively contribute to better cognitive outcomes in various populations (Bettio et al., 2020; Donnelly et al., 2016; Hueston et al., 2017; A. Li et al., 2019).

However, the effectiveness of physical activity in promoting cognitive performance and learning outcomes is not solely dependent on the physiological processes that occur within the body during physical activity. Emerging research suggests that environmental factors, collectively known as the exposome, play a significant role in shaping the effects of physical activity on cognitive function (Gorman et al., 2021; Lee et al., 2024; McGaughey et al., 2020; Zhao et al., 2025). The exposome encompasses the totality of environmental exposures an individual experiences over their lifetime, including both external factors such as air pollution, noise, and access to green spaces, and internal factors such as psychological stress. In urban environments, the exposome can have a profound impact on the cognitive benefits derived from physical activity, particularly in terms of how environmental stressors influence neurocognitive processes (Devarajan et al., 2020; Robinson et al., 2025; Sallis et al., 2016; Z. Wang et al., 2025).

The concept of the exposome is relatively new but is rapidly gaining traction in environmental health and public health research. The term was first coined by Wild (2005) to describe the cumulative environmental factors that influence human health. These factors include everything from air and water quality to the built environment and psychosocial stressors. The urban exposome, in particular, refers to the specific environmental exposures that individuals encounter in urban settings, such as air pollution, noise, and limited access to green spaces (Vrijheid, 2014). Urban environments are typically associated with high levels of pollution, noise, and overcrowding, all of which can negatively impact physical and mental health. Moreover, urbanization often limits access to natural environments, which in turn has been associated with negative impacts on both physical health and cognitive performance (Vineis et al., 2020).

In this context, it is essential to consider how urban environmental stressors interact with physical activity to influence cognitive outcomes, particularly in terms of learning. Learning is a multifaceted process that involves various cognitive functions such as attention, memory, executive control, and processing speed (Estes, 2022). Physical activity has been shown to enhance all of these cognitive functions, likely through mechanisms such as increased brain-derived neurotrophic factor (BDNF) levels (Latomme et al., 2022), improved blood flow to the brain (Kleinloog et al., 2022), and the regulation of neurotransmitters (Bhattacharya et al., 2023). However, environmental factors like air pollution and noise can hinder these benefits, limiting the positive impact of physical activity on cognitive outcomes (Walsh et al., 2020).

### 1.1. Cognitive and Educational Implications of Physical Activity in Urban Environments

The role of environmental stressors in shaping the cognitive benefits of physical activity is particularly important in educational settings (Byshevets et al., 2024; Morsanuto et al., 2023; Strehli et al., 2021). School-aged children and adolescents, who are at a critical stage of cognitive and emotional development, are especially vulnerable to the negative effects of the urban exposome (Greco et al., 2019; Moore et al., 2022). Many schools are located in urban areas where pollution and noise are prevalent, and access to green spaces is often limited. The lack of natural environments in these settings may attenuate the positive effects of physical activity on learning, resulting in suboptimal educational outcomes for students (Juneja, 2021; Pfledderer et al., 2021). As such, understanding the interaction between physical activity, the urban exposome, and cognitive performance in educational contexts is crucial for developing effective strategies to improve learning outcomes (Mitchell, 2013).

Previous research has examined the effects of physical activity on cognitive performance in various populations, including children, adults, and older adults (Erickson et al., 2022; Ferreira Vorkapic et al., 2021; García-Hermoso et al., 2021; Gavelin et al., 2021). In general, studies have consistently shown that physical activity has a positive impact on cognitive function, particularly in terms of attention, memory, and executive function (Sibbick et al., 2022; Singh et al., 2025). Regular physical activity has been shown to improve working memory, increase the ability to concentrate, and enhance problem-solving skills (Srinivas et al., 2021). These benefits are thought to arise from the physiological changes that physical activity and exercise induce in the brain, including the release of neurotrophic factors, such as BDNF, which support the growth and survival of neurons, and improvements in brain plasticity, which enhance the brain’s ability to adapt and reorganize in response to new information (Bidzan-Bluma & Lipowska, 2018).

However, environmental factors can significantly influence the magnitude of these benefits. For instance, exposure to high levels of air pollution has been shown to impair cognitive function and reduce the benefits of physical activity. Studies have found that air pollution negatively affects brain development in children and accelerates cognitive decline in older adults (Chandra et al., 2022). Similarly, chronic exposure to noise has been linked to increased stress levels, which can hinder cognitive performance and learning (Manohar et al., 2022). Stress is known to have a particularly detrimental effect on cognitive functions such as memory and attention, and environmental stressors such as noise and pollution can exacerbate the physiological and psychological stress responses, thus reducing the cognitive benefits of physical activity (Walters et al., 2025).

In contrast, access to green spaces and natural environments has been shown to enhance cognitive performance, particularly in urban areas where access to nature is often limited (Vella-Brodrick & Gilowska, 2022). Nature-based interventions, such as physical activity in parks or green spaces, have been associated with improved attention, working memory, and mood. Studies have demonstrated that even brief exposure to natural environments can reduce stress and improve cognitive performance, suggesting that the presence of green spaces in urban settings may enhance the positive effects of physical activity on cognitive function (Harada et al., 2016).

### 1.2. Research Rationale and Hypotheses

Given the growing body of research on the relationship between physical activity, the urban exposome, and cognitive function, there is a need to better understand how these factors interact to influence learning outcomes in urban populations (Zijlema et al., 2017). Specifically, how do environmental stressors such as air pollution, noise, and limited access to green spaces modulate the cognitive benefits of physical activity? How can physical activity be optimized in urban environments to enhance cognitive performance and learning in adolescents? These questions are of critical importance in the context of educational settings, where physical activity is often integrated into the curriculum to promote learning and well-being.

This study aimed to address these questions by exploring the role of the urban exposome in modulating the cognitive benefits of physical activity. By examining the effects of physical activity in different urban environments, including green parks, high-traffic streets, and controlled indoor spaces, this research sought to provide a comprehensive understanding of how environmental factors influence cognitive outcomes following physical activity. The findings of this study have important implications for urban planning, school-based physical education programs, and public health interventions aimed at improving both physical and cognitive health in urban populations. Through this investigation, we aimed to contribute valuable insights into how the urban exposome can be leveraged to optimize the cognitive benefits of physical activity and improve learning outcomes in educational contexts.

We hypothesized that (1) adolescents performing physical activity in natural or semi-natural urban environments would exhibit greater improvements in cognitive performance and stress reduction compared to those exercising in controlled indoor settings; and (2) environmental variability, particularly exposure to green spaces, would positively moderate the relationship between physical activity and cognitive outcomes.

## 2. Materials and Methods

### 2.1. Study Design

This study employed a multi-method experimental design (Johnson & Christensen, 2017), combining several quantitative measures to assess cognitive, psychological, and physiological responses to physical activity in different urban environments. This approach allowed for a comprehensive evaluation of the effects of environmental variability through multiple instruments (cognitive tests, perceived stress scales, and physiological monitoring) within a unified experimental framework. Participants were randomly assigned to one of two groups: an experimental group and a control group, in order to compare the effects of various urban environments on physical activity and cognitive performance. The parallel groups approach meant that participants in both groups followed separate paths during the intervention, with measurements taken before and after each physical activity session. The experimental group exercised in varying urban environments (parks, busy streets, indoor spaces), while the control group followed a physical activity program in a neutral and controlled environment, free from exposure to external environmental stressors. The study was conducted in accordance with the ethical guidelines for research with human beings established by the Declaration of Helsinki. The study was conducted from November 2024 to March 2025. It was approved by the Department of Medical, Motor, and Wellbeing Sciences of the University of Naples “Parthenope” (DiSMMeB Prot. N. 88592/2024). Informed consent was obtained from the parents of all participants, as they were minors, and participants were informed of the possibility to withdraw from the study at any time, without any negative consequences.

### 2.2. Participants

The study included a sample of 60 adolescent participants, divided into two groups: the experimental group and the control group, each consisting of 30 participants. Participants were recruited from three local secondary schools in the Naples metropolitan area through collaboration agreements with school administrations and physical education teachers. Participants were recruited from a population of young adolescents aged between 15 and 18 years, in good health, and with a history of regular physical activity. The selection of participants was based on specific inclusion criteria to ensure sample homogeneity and the safety of participants during physical activity. Participants were included in the study if they met specific eligibility criteria. They were required to be adolescents between 15 and 18 years of age and in good general health, without any medical conditions that could interfere with physical activity or with their participation in the study. Eligible participants also had a documented history of regular moderate physical activity, engaging in such activities at least two to three times per week for a minimum of three months prior to recruitment. In addition, all participants and their legal guardians provided written informed consent before enrollment in the study, in accordance with ethical research standards. Exclusion criteria were established to ensure participant safety and data reliability. Individuals presenting neurological or psychiatric disorders, such as cognitive impairments, depression, anxiety, or psychotic conditions, were not eligible for participation. Similarly, those with uncontrolled cardiovascular problems, including untreated heart disease or hypertension, were excluded to prevent potential health risks during the physical activity sessions. Participants who were taking medications known to affect physiological responses to physical activity or cognitive functioning (e.g., anxiolytics or antidepressants) were also excluded. Finally, individuals who were unable to commit to the full duration of the intervention or to attend sessions regularly were not included in the final sample.

Before starting the study, an a priori analysis was conducted using G*Power version 3.1.9.7 software to determine the minimum number of participants required. The analysis suggested that, with a significance level of 0.05, a statistical power of 80%, and a medium effect size (Cohen’s d = 0.5), the minimum number of participants per group should be 30. Therefore, 60 participants were recruited in total, equally divided between the experimental and control groups. The final sample consisted of participants who met the inclusion and exclusion criteria. The participants were distributed between the experimental and control groups, and their characteristics are summarized in the following table (Table 1):

Each participant was subjected to pre- and post-intervention measurements to monitor the effects of physical activity on cognitive performance and stress levels. The randomization of participants into the experimental and control groups ensured that any differences in outcomes could be attributed to the effects of the urban environment (parks, busy streets, indoor spaces) or the controlled environment of the control group. This approach allowed for the isolation of the effect of physical environments on cognitive outcomes related to learning.

### 2.3. Procedures

Participants were recruited through local advertisements and invitations to take part in a study on the benefits of physical activity. After providing informed consent, participants underwent a preliminary screening to ensure they met the inclusion criteria established by the study.

Participants were randomly assigned to either the experimental or control group. This randomization ensured the absence of bias in participant distribution and minimized differences between groups, allowing the isolation of the effects of urban environments on physical activity and cognitive performance.

Participants in both groups performed moderate-intensity physical activity (approximately 60–70% of maximum heart rate) for a duration of 60 min, three times a week for two weeks. All sessions took place at the same time of day, between 3:00 and 4:00 p.m., immediately after school hours, to ensure physiological consistency and minimize circadian influences. For outdoor sessions, activities were carried out only under comparable weather conditions, avoiding extreme temperatures or precipitation. In the event of adverse weather, sessions were moved to a covered urban courtyard to maintain the same timing and conditions. Each session was supervised and facilitated by the same exercise specialist, a graduate in Sports and Exercise Science, from the research team, assisted by two researchers responsible for monitoring and supervising the sessions. The physical activity protocol was conducted under strictly controlled and standardized conditions, and participants were instructed to focus on the activity without engaging in conversation, to minimize potential social interaction effects. During each session, participants in the experimental group exercised in one of three environments: urban park, busy street, or indoor gym. In contrast, the control group performed the same exercises in a controlled indoor environment, free from external environmental factors that could influence the results.

Measures were collected before and after each exercise session to assess changes in cognitive performance, perceived stress levels, and physiological responses. All pre- and post-tests were administered under the direct supervision of the research team in a quiet and controlled environment immediately before and after each session. Participants completed the assessments simultaneously, each working individually on their assigned materials or device under researcher supervision. The Stroop Test was administered in a computer-based version to ensure accuracy in response timing, whereas the Perceived Stress Scale (PSS) was completed in a paper-and-pencil format.

Before and after each session, participants underwent a series of cognitive tests to assess different dimensions of cognitive function. These tests included: (i) Short-term memory test (Digit Span); (ii) Sustained attention test (Continuous Performance Test—CPT); (iii) Executive function test (Stroop Test).

Perceived stress levels were measured using the Perceived Stress Scale (PSS), administered both before and after each exercise session, to assess the impact of urban environments on psychological stress indicators.

Heart Rate Variability (HRV) was monitored to measure the physiological response to exercise and how it might have reflected acute stress caused by different urban environments. HRV is an important indicator of autonomic regulation and the balance between the sympathetic and parasympathetic nervous systems, providing information about stress levels and physiological recovery after physical activity.

### 2.4. Measures

#### 2.4.1. Digit Span Test

The Digit Span (Woods et al., 2011) was used to assess short-term memory and attention capacity in participants, which are crucial cognitive aspects that may be influenced by environmental stress during physical activity. This test allowed us to examine how different urban environments might impact these cognitive functions during the physical activity program. In the test, participants were presented with a sequence of numbers that they had to repeat in the correct order (Digit Span forward) or in reverse order (Digit Span backward). The difficulty of the test increases as the length of the sequence grows. The final score was determined by the longest sequence that the participant could repeat correctly in both conditions. Each part of the test was repeated multiple times to ensure accurate measurement of memory and attention capacities. The test was administered orally, with the participant asked to repeat the numbers immediately after hearing them. Each session lasted approximately 5–10 min, and the Digit Span Test was administered before and after each exercise session to monitor changes in short-term memory and attention capacity over time.

#### 2.4.2. Continuous Performance Test (CPT)

The Continuous Performance Test (CPT) (Rahman et al., 2022) was administered to assess sustained attention and concentration capacity of the participants during physical activity. This test was chosen to investigate how exposure to different urban environments, such as busy streets or green spaces, might influence participants’ ability to maintain attention over extended periods. In the CPT, participants were exposed to visual or auditory stimuli through a computer and had to press a key whenever a target stimulus appeared. The stimuli were presented in continuous sequences, and the participant had to respond only to the target stimuli while ignoring the others. The score was calculated based on the percentage of correct responses, average reaction time, omission errors (failure to respond to target stimuli), and commission errors (incorrect responses to non-target stimuli). The test was performed in a controlled environment for 15–20 min, with participants instructed to maintain high levels of attention and respond promptly. The CPT was administered before and after each exercise session to evaluate potential changes in sustained attention and concentration across the intervention period.

#### 2.4.3. Stroop Test

The Stroop Test (Kestens et al., 2021) was used to assess executive function, particularly inhibitory control and selective attention. In the context of this study, the test allowed us to evaluate how participants responded to situations requiring them to inhibit automatic responses (such as reading a word) in order to focus on a different task (for example, naming the color of ink in which the word is written). This was particularly relevant for investigating the effect of urban environments on cognitive abilities during physical activity. In the test, participants had to name the color in which the words were written (e.g., the word “red” written in green), ignoring the meaning of the word. The final score was determined by the number of correct responses given within a set time frame, with a separate evaluation for each of the three conditions: color words, colors, and the interference condition. Each part of the test lasted approximately 45 s, and the total administration time was around 5–10 min. The Stroop Test was administered before and after each exercise session to monitor potential changes in executive function and inhibitory control across the intervention period.

#### 2.4.4. Perceived Stress Scale (PSS)

The Perceived Stress Scale (PSS) (Yılmaz Koğar & Koğar, 2024) was administered to measure participants’ perceived stress levels before and after each exercise session. This measure was used to explore the effect of urban environments on the participants’ psychological stress during physical activity. The PSS is a 10-item scale that assesses how participants perceived stress over the past few weeks, using a scale from 0 to 4 (where 0 means “never” and 4 means “very often”). The final score was obtained by summing the responses. A higher score indicates a higher perceived level of stress. The PSS was administered both before and after each exercise session, allowing us to track any changes in stress levels related to the different urban environments. The administration duration was about 5–10 min.

#### 2.4.5. Heart Rate Variability (HRV)

Heart Rate Variability (HRV) (Pham et al., 2021) was monitored to measure participants’ physiological response to physical activity and how it might reflect acute stress caused by different urban environments. HRV is an important indicator of autonomic regulation and the balance between the sympathetic and parasympathetic nervous systems. In this study, HRV was monitored using a wearable sensor, which allowed for real-time data collection during each exercise session. Participants were provided with a wearable device that monitored heartbeats and calculated the variation between beats. HRV was used to assess the physiological response to stress and the recovery capacity after exercise. Greater heart rate variability indicates better recovery and a more favorable autonomic balance. HRV data were collected throughout the entire exercise session, which lasted 60 min for each session.

### 2.5. Physical Activity Intervention Program

The physical activity intervention was designed to last 12 weeks, with three exercise sessions per week. The program was structured for both groups: the experimental group and the control group, with the only difference being that the experimental group performed exercises in variable urban environments, while the control group performed the same program in a controlled indoor environment, free from external environmental stressors. The main objective of the intervention was to examine the effects of physical activity on cognitive performance, perceived stress, and physiological responses in relation to different urban contexts. The duration and frequency of the program were based on established evidence indicating that moderate-intensity exercise performed three times per week for at least 12 weeks can enhance cognitive performance, executive function, and stress regulation (Nakagawa et al., 2020; Pesce et al., 2021; Tao et al., 2025; H. Wang et al., 2023). This structure was therefore chosen to align with prior research demonstrating the cognitive and psychological benefits of consistent, moderate-intensity physical activity.

**Weeks 1–4: Adaptation and Building Endurance**

During the first 4 weeks, the primary goal was to introduce the participants to physical activity and prepare the body for moderate intensity sessions. The exercises were designed to improve cardiovascular endurance, overall muscle strength, and motor coordination.

Exercise Program for Weeks 1–4:

- Warm-Up (10 min):
  ○ Brisk walking or light jogging: Performed for 5 min, followed by dynamic stretching (approximately 5 min) to prepare muscles and improve joint mobility.
- Aerobic Exercises (30 min):
  ○ Brisk walking or light jogging: 15 min of brisk walking or light jogging.
  ○ Stationary bike or treadmill for the control group, and equivalent walking or cycling activities performed outdoors in parks for the experimental group: 15 min of cycling or walking on a treadmill or cycle ergometer for the control group.
- Strength Exercises (15 min):
  ○ Squats (3 sets of 12 repetitions).
  ○ Lunges (3 sets of 12 repetitions per leg).
  ○ Push-ups (3 sets of 10–15 repetitions).
  ○ Planks (3 sets of 20 s each).
- Cool-Down (5 min):
  ○ Static stretching to relax muscles and improve flexibility.

**Weeks 5–8: Increased Intensity and Greater Resistance Work**

Starting from the fifth week, the intensity of the exercise was increased to stimulate further improvement in aerobic capacity and to enhance muscle strength. The exercises were designed to focus more on improving endurance and power, with greater emphasis on strength training.

Exercise Program for Weeks 5–8:

- Warm-Up (10 min):
  ○ Light running or cycling: 5 min of light running or low-intensity cycling, followed by dynamic stretching.
- Aerobic Exercises (35 min):
  ○ Moderate running (or jogging): 20 min of moderate running for the control group; the experimental group alternated between running or walking in various urban environments, such as parks or busy streets, to examine the impact of different environmental stimuli.
  ○ Intermittent effort: 5 min of jogging alternating with 1 min of intense running, followed by 1 min of walking, for the control group.
- Strength Exercises (15 min):
  ○ Bodyweight squats (4 sets of 12 repetitions).
  ○ Lunges in motion (4 sets of 12 repetitions per leg).
  ○ Push-ups (4 sets of 12 repetitions).
  ○ Planks (4 sets of 30 s each).
- Cool-Down (5 min):
  ○ Static stretching (5 min to improve flexibility and reduce the risk of injury).

**Weeks 9–12: Optimization and Enhancement**

In the final 4 weeks, the program reached its peak intensity, with the goal of further improving aerobic endurance, muscle strength, and overall performance. The exercises focused on more complex movements and high-intensity workouts to stimulate greater physiological adaptability.

Exercise Program for Weeks 9–12:

- Warm-Up (10 min):
  ○ Moderate running or cycling with increasing intensity: 5 min of light running, followed by dynamic stretching (5 min) to prepare muscles and joints.
- Aerobic Exercises (40 min):
  ○ Running at moderate to high intensity (control group)/alternating between parks and busy streets (experimental group): 20 min of progressively intense running.
  ○ Circuit of aerobic and anaerobic exercises: 10 min of interval training, with 1 min of high-intensity running, followed by 1 min of walking or active rest.
  ○ High-intensity functional exercise: 10 min of functional training, including exercises like mountain climbers, jumping jacks, and burpees.
- Strength Exercises (15 min):
  ○ Squats with load (5 sets of 12 repetitions).
  ○ Weighted lunges (5 sets of 12 repetitions per leg).
  ○ Push-ups with variations (narrow or wide hand placement) (5 sets of 12 repetitions).
  ○ Side planks (5 sets of 30 s per side).
- Cool-Down (5 min):
  ○ Static stretching for the whole body to promote muscle relaxation and reduce the risk of injury.

For the experimental group, the exercise was performed in three different urban environments each week: urban parks (green spaces), busy streets (polluted environments), and covered urban courtyard. These environments were chosen to examine how various urban stimuli (such as air pollution, noise, and visual variety) could influence the physiological and cognitive response of individuals during physical exercise. The exercise sessions combined aerobic and strength-based activities designed to be adaptable across all urban environments. Aerobic exercises included brisk walking, jogging, and stair climbing, while strength exercises consisted of bodyweight movements such as squats, lunges, and push-ups, which could be safely performed in outdoor areas (e.g., parks and wide sidewalks) as well as in indoor environments. The same standardized equipment (elastic resistance bands and light dumbbells) was used in all settings, including outdoor locations, to ensure consistency in exercise intensity and type. No treadmills were used in the outdoor sessions, as activities were performed on natural terrain or pavement under researcher supervision. The control group performed the same exercises in a controlled indoor environment, free from external environmental factors.

### 2.6. Statistical Analysis

In this study, a variety of statistical tests were employed to assess the effectiveness of the physical activity intervention on cognitive performance, stress levels, and physiological responses. The primary aim was to analyze the changes in measurements taken before and after the intervention, comparing both within-group variations and differences between the two groups.

To begin, paired samples *t*-tests were conducted to compare the pre- and post-intervention scores within each group, both experimental and control. This test was essential to determine if significant changes occurred in cognitive performance, perceived stress, and physiological responses following the physical activity intervention. Specifically, it was used to analyze changes in areas such as working memory (Digit Span), attention (Continuous Performance Test—CPT), cognitive flexibility (Stroop Test), and perceived stress (measured by the Perceived Stress Scale—PSS).

In addition to the within-group comparisons, independent samples *t*-tests were employed to compare the post-intervention results between the experimental and control groups. This test helped to determine whether the different environments, such as urban parks, busy streets, or neutral indoor gyms, had a differential effect on cognitive performance, perceived stress levels, and heart rate variability (HRV). By comparing the two groups at the post-test stage, we could evaluate the impact of the physical activity intervention across different environmental settings.

Moreover, to better understand the magnitude of the observed differences, effect size calculations were performed using Cohen’s d. This measure provided valuable insight into the practical significance of the results, beyond the statistical significance indicated by *p*-values. Effect size helps quantify how large the difference is between the pre- and post-intervention results and between the experimental and control groups, offering a more detailed understanding of the intervention’s impact.

In addition to the above tests, ANOVA and Pearson’s correlations were also conducted. ANOVA (Analysis of Variance) was used to assess any potential interaction effects or differences between more than two groups, allowing us to explore whether the varying urban environments significantly influenced the outcomes. Pearson’s correlation was employed to examine the relationships between cognitive performance and perceived stress, further elucidating any connections between the psychological and physiological responses to exercise in different environments.

Finally, a significance level of 0.05 was used for all tests. This threshold ensured that the observed differences were statistically significant and unlikely to have occurred by chance, helping to reinforce the robustness of the findings. For the statistical calculations, SPSS (Statistical Package for the Social Sciences—IBM Corp., Armonk, NY, USA), version 27.0, was used.

These statistical methods, when combined, provided a comprehensive framework for evaluating the effects of physical activity in different urban environments. The use of paired samples *t*-tests for within-group comparisons, independent samples *t*-tests for between-group differences, along with effect size calculations, ANOVA, and correlations, helped ensure a robust and thorough analysis of how the physical activity intervention impacted cognitive performance, stress levels, and physiological responses.

## 3. Results

### 3.1. Paired t-Test

The paired *t*-test (Table 2) was used to analyze the effects of physical activity in various urban environments on memory, attention, and perceived stress levels. The results indicate a significant improvement in all three measured variables in the experimental group, as evidenced by the statistical tests performed. All t-values are significant with *p* < 0.05 (Table 2), suggesting a significant improvement in cognitive performance (memory, attention, and executive functions) and a significant reduction in stress levels in the experimental group following the physical activity intervention. Additionally, heart rate variability (HRV) showed an improvement, indicating a positive physiological response to exercise.

### 3.2. ANOVA

The two-way repeated measures ANOVA was conducted to assess the effects of the physical activity intervention on five key variables: Digit Span Test (memory), Continuous Performance Test (CPT, attention), Stroop Test (cognitive flexibility), Perceived Stress Scale (PSS), and Heart Rate Variability (HRV), both within the experimental and control groups. This statistical test was designed to examine both the main effects (i.e., the effect of time and group) and interaction effects (i.e., the combined effect of time and group).

The main effects for each variable were tested, which included comparing the pre-intervention and post-intervention scores within each group. The results revealed that for all five variables, there were significant changes over time, specifically in the experimental group. In other words, for each of these variables, the difference between pre- and post-intervention was statistically significant in the experimental group, suggesting that the physical activity intervention had a notable impact on cognitive performance, perceived stress, and physiological responses (Table 3).

The interaction effect in the ANOVA refers to how the time factor (pre- vs. post-intervention) interacted with the group factor (experimental vs. control). The results revealed that the changes observed in the experimental group were significantly greater than those observed in the control group. This suggests that the intervention had a greater impact on the experimental group, where improvements in cognitive performance, stress reduction, and HRV were more pronounced compared to the control group. In contrast, the control group exhibited only minor or non-significant improvements between pre- and post-intervention measurements, confirming that the absence of environmental variation resulted in less pronounced effects compared with the experimental group.

### 3.3. Independent t-Test

The independent *t*-test results (Table 4) compared post-intervention outcomes between the experimental group (participants exercising in variable urban environments) and the control group (participants exercising in a controlled indoor environment). The analysis revealed significant improvements across all tested variables, including cognitive performance (memory, attention, and cognitive flexibility), stress reduction, and physiological health (HRV) in favor of the experimental group. This suggests that the physical activity intervention, particularly within varied urban environments, had a positive effect on both cognitive and physiological outcomes, supporting the existing body of literature on the benefits of physical activity for mental and physical health.

### 3.4. Pearson Correlations

**Figure 1:**
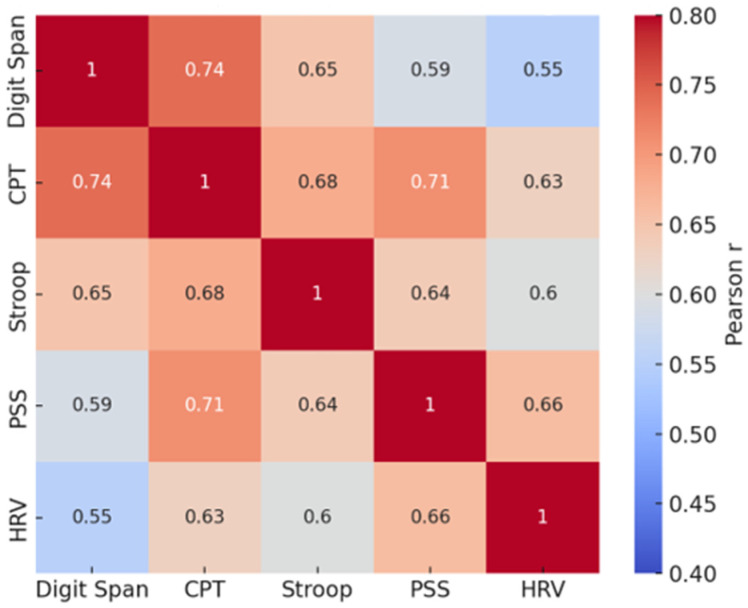
Pearson correlation results with the correlation matrices for the experimental group.

Pearson’s correlations were calculated among the post-intervention scores to examine the relationships between physiological and cognitive outcomes. The results revealed moderate-to-strong positive associations between perceived stress (PSS) and heart rate variability (HRV) (r = 0.63), as well as between HRV and cognitive performance measures such as the Continuous Performance Test (CPT) (r = 0.71) and Stroop Test (r = 0.68). Working memory performance, as measured by the Digit Span test, was moderately correlated with CPT (r = 0.74) and Stroop (r = 0.65) scores. These correlations suggest that improvements in autonomic regulation were associated with enhanced attention and cognitive flexibility after the intervention (Figure 1).

## 4. Discussion

This study aimed to investigate the effects of physical activity in different urban environments on cognitive performance, stress levels, and physiological responses. The primary objectives were to determine whether physical activity performed in diverse urban contexts (such as urban parks, busy streets, and neutral indoor gyms) could influence cognitive functions, perceived stress, and heart rate variability (HRV) in adolescents. In addition, we sought to examine whether these effects were significantly greater in the experimental group (exercising in varying urban environments) compared to the control group (exercising in a controlled indoor environment).

Our results suggest that the experimental group showed significant improvements in all the assessed variables: cognitive performance (including memory, attention, and cognitive flexibility), perceived stress, and physiological responses (as measured by HRV). The control group, on the other hand, showed modest within-group improvements, but these changes were not statistically significant when compared with the experimental group.

Although the study did not aim to compare specific urban settings, preliminary observations did not reveal evident differences among the park, busy street, and covered urban courtyard environments. This suggests that the beneficial effects observed may derive from the overall exposure to environmental variability rather than a single type of urban context.

### 4.1. Cognitive, Psychological, and Physiological Outcomes

The results from the cognitive performance tests revealed significant improvements in the experimental group across all domains assessed: memory (measured by the Digit Span test), attention (Continuous Performance Test—CPT), and cognitive flexibility (Stroop Test). These improvements can be attributed to the combination of physical activity and exposure to diverse urban environments, which may have stimulated various aspects of cognitive functioning.

The improvement in memory, particularly in working memory as assessed by the Digit Span test, is consistent with previous studies that highlight the positive effects of physical activity on cognitive functions. Regular physical activity has been shown to enhance neuroplasticity, the brain’s ability to form and reorganize synaptic connections, thus improving cognitive processes such as memory (Erickson et al., 2022; Kim & Sung, 2017; Liang et al., 2021). Physical activity, especially when performed in novel or stimulating environments, may also engage different neural networks involved in memory consolidation and retrieval (Duzel et al., 2016). In our study, the exposure to varying urban environments likely provided novel and dynamic sensory experiences that may have further enhanced the cognitive benefits of physical activity, as environmental complexity has been linked to cognitive stimulation (Berman et al., 2008).

The significant improvement in attention, as measured by the Continuous Performance Test (CPT), also aligns with findings from previous research. Studies have demonstrated that physical activity can enhance selective attention, the ability to focus on relevant stimuli while ignoring distractions (Hillman et al., 2008). The interaction between physical activity and the varied sensory stimuli in urban environments may have contributed to an enhanced capacity for sustained attention (Walters et al., 2025). Urban environments, particularly those involving moderate levels of complexity (such as parks or busy streets), may offer a level of sensory input that challenges and therefore strengthens attentional control (Han, 2021). Moreover, the controlled physical activity condition, where participants worked out in a neutral indoor setting, lacked the external stimuli that could have engaged and trained attentional resources. This could explain why the experimental group exhibited greater improvements in attention compared to the control group, which only engaged in physical activity within a stable, predictable environment.

Cognitive flexibility, as measured by the Stroop Test, is another key cognitive function that showed improvement in the experimental group. Cognitive flexibility refers to the ability to switch between different tasks or mental sets and adapt to new and unexpected information. This ability is crucial in daily life, especially in environments that constantly demand mental flexibility (Canas et al., 2006). The significant improvement in cognitive flexibility in the experimental group can be explained by the fact that urban environments are often unpredictable, requiring individuals to constantly adapt to new stimuli and information (Cañas et al., 2003). This type of dynamic environment may have provided the necessary challenge to enhance cognitive flexibility, as people are forced to adjust their responses to fluctuating surroundings (Gates & Valenzuela, 2010).

Research suggests that physical activity in environments that vary in complexity and sensory stimulation may act as a form of cognitive training, improving individuals’ capacity to process and switch between different cognitive tasks (Di Palma et al., 2025; Hannan, 2014; Kashihara et al., 2009). In this study, the diversity of the urban environments exposed participants to different cognitive challenges, which likely contributed to enhanced performance on the Stroop test.

Perceived stress was significantly reduced in the experimental group, as measured by the Perceived Stress Scale (PSS). This finding supports the hypothesis that physical activity in natural or semi-natural environments can lead to greater reductions in stress levels compared to exercise in controlled environments (Yao et al., 2021). Urban parks and green spaces have long been associated with stress-reducing effects (Iso-Markku et al., 2024), and our results align with these studies. The physical activity, combined with the exposure to natural elements, likely triggered physiological and psychological processes that led to stress relief (Yeh et al., 2016).

Several mechanisms may account for this effect. First, physical activity itself is well-known for its stress-relieving properties, as it promotes the release of endorphins and other neurochemicals that improve mood and reduce anxiety (Yang et al., 2024; Scamardella et al., 2020a, 2020b). Second, exposure to natural environments, even in urban settings, has been shown to lower cortisol levels and promote relaxation (X. Li & Li, 2025). Our findings suggest that the combination of these factors in the experimental group led to a greater reduction in perceived stress compared to the control group, which did not experience the same level of environmental variation or natural stimuli during physical activity.

Heart rate variability (HRV), which reflects the balance between the sympathetic and parasympathetic nervous systems, was also significantly higher in the experimental group. HRV is a key indicator of autonomic nervous system regulation and is considered a marker of physical health and resilience to stress (Fernández-Jiménez et al., 2024). A higher HRV generally indicates better adaptability to stress and a more relaxed physiological state.

The positive impact of physical activity on HRV is well-documented, as regular physical activity has been shown to enhance autonomic regulation (Teuber et al., 2024). In this study, the experimental group exhibited an increase in HRV after physical activity, which could be due to both the physical activity itself and the exposure to varying environmental stimuli. physical activity in complex, dynamic environments may have further challenged the autonomic system, leading to improved regulatory capacity. The control group, which exercised in a neutral, controlled indoor environment, did not experience the same level of environmental complexity and thus did not show the same degree of improvement in HRV.

### 4.2. Limitations and Future Directions

While the results of this study are promising, it is important to consider several limitations. First, the study’s duration was limited to only 12 weeks, and the long-term effects of physical activity in urban environments on cognitive performance and stress remain unclear. Future research should examine the sustainability of these effects over longer periods. Second, while we controlled for factors such as age and baseline fitness, individual differences in response to environmental stressors and physical activity may have influenced the outcomes. It would be valuable for future studies to explore how individual characteristics, such as personality traits or baseline stress levels, interact with environmental factors to shape the benefits of physical activity.

### 4.3. Practical Implications

Despite these limitations, the findings of this study have important implications for urban planning and public health. Our results suggest that urban environments can play a crucial role in enhancing the cognitive and psychological benefits of physical activity. Incorporating more green spaces and areas with varied sensory stimuli in urban areas could have a positive impact on public health by promoting cognitive function, reducing stress, and improving overall well-being.

In light of the discussion, the results of this study highlight the significant benefits of physical activity in varied urban environments on cognitive performance, stress reduction, and physiological responses. The experimental group, which exercised in dynamic urban settings such as parks and busy streets, showed marked improvements in cognitive functions, reduced perceived stress, and improved HRV. These findings align with existing literature on the positive effects of physical activity and exposure to natural environments on cognitive and psychological well-being. The combination of physical activity and environmental variability may provide a powerful tool for improving mental and physical health, with implications for future urban development and public health interventions.

Further research is needed to confirm the long-term effects of these interventions and to explore the mechanisms behind these observed benefits.

## 5. Conclusions

In recent years, the understanding of how urban environments impact physical activity and cognitive performance has gained significant attention, particularly in relation to how various stressors in these environments may influence the overall effectiveness of physical activity interventions. The present study aimed to explore the effects of different urban environments on cognitive performance, stress levels, and physiological responses during moderate-intensity physical activity.

This study provided valuable insights into the effects of urban environments on physical activity outcomes, specifically cognitive performance, stress reduction, and physiological responses. The results indicated that participants in the experimental group, who performed physical activity in diverse urban settings, exhibited significant improvements in cognitive functions, such as memory, attention, and executive functions, compared to the control group, which exercised in a neutral indoor environment and showed only modest or non-significant improvements across the same variables. Additionally, these improvements were associated with a marked reduction in perceived stress levels and enhanced heart rate variability (HRV), suggesting a beneficial impact of physical activity in real-world environments.

Our findings support the idea that urban spaces, including parks, busy streets, and indoor gyms, provide unique stimuli that can influence both physical and mental well-being during physical activity. These results contribute to a growing body of evidence suggesting that the combination of physical activity and environmental factors plays a crucial role in enhancing cognitive functions and managing stress. Moreover, the increased HRV observed in the experimental group further emphasizes the physiological benefits of exercising in environments that challenge the body and mind.

The study also underscores the importance of considering environmental factors when designing physical activity interventions. While controlled indoor environments are often used in research and clinical settings, the real-world complexity of urban environments might offer more dynamic and stimulating conditions that can optimize the benefits of physical activity.

Further research is necessary to explore the long-term effects of exercising in different urban environments and to determine the specific mechanisms through which these environments influence cognitive and physiological outcomes. Future studies could also investigate the impact of different types of exercise (e.g., strength training, aerobic exercise) on the same cognitive and physiological measures in various environmental contexts.

In conclusion, this study highlights the potential of incorporating urban environments into physical activity interventions as a means of promoting both physical and mental health. The findings suggest that physical activity in such settings may offer greater cognitive and stress-related benefits compared to more controlled physical activity environments, emphasizing the need for future research to further investigate the complex interactions between exercise, urban environments, and health outcomes.

## Figures and Tables

**Table 1:** Characteristics of Participants in the Experimental and Control Groups.

Characteristic	Experimental Group (30 Participants)	Control Group (30 Participants)
Number of participants	30	30
Age (mean ± SD)	16.5 ± 5.2 years	16.3 ± 4.8 years
Gender (male/female)	15 males, 15 females	14 males, 16 females
Health status	Good health, no severe medical conditions	Good health, no severe medical conditions
Physical activity (weekly frequency)	2–3 sessions of moderate physical activity weekly	2–3 sessions of moderate physical activity weekly
Exclusion criteria	Neurological/psychiatric disorders, uncontrolled cardiovascular diseases	Neurological/psychiatric disorders, uncontrolled cardiovascular diseases
Type of exercise	Physical activity in variable urban environments (parks, busy streets, covered urban courtyard)	Physical activity in a controlled environment (indoor gym)

**Table 2:** Effects of physical activity on cognitive performance and stress indicators (Paired *t*-test within groups and post-intervention between-group comparison).

Variable	EG Pre (Mean ± SD)	EG Post (Mean ± SD)	t-Value (EG)	*p*-Value (EG)	CG Pre (Mean ± SD)	CG Post (Mean ± SD)	t-Value (CG)	*p*-Value (CG)	Post-Intervention Comparison (EG vs. CG) t-Value	*p*-Value
Digit Span Test	15 ± 2.6	18 ± 2.5	5.23	0.00002	15 ± 2.3	16 ± 2.0	1.45	0.175	3.18	0.003
CPT	43 ± 5.2	48 ± 5.5	4.75	0.0001	42 ± 5.0	44 ± 4.8	1.12	0.265	2.52	0.014
Stroop Test	18 ± 3.0	23 ± 3.1	6.12	0.00003	19 ± 2.8	21 ± 2.7	1.30	0.210	2.14	0.037
PSS	21 ± 2.1	16 ± 2.0	−7.35	0.00001	19 ± 2.4	18 ± 2.3	−0.85	0.402	−3.62	0.001
HRV	34 ± 4.9	40 ± 5.0	4.90	0.00005	35 ± 4.5	36 ± 4.2	0.78	0.460	2.52	0.014

**Table 3:** Two-way repeated measures ANOVA results.

Variable	F-Value	*p*-Value	Significant Change
Digit Span Test (Memory)	8.34	<0.05	Yes
CPT (Attention)	14.52	<0.05	Yes
Stroop Test (Flexibility)	12.56	<0.05	Yes
PSS (Stress)	6.12	<0.05	Yes
HRV (Heart Rate Variability)	9.47	<0.05	Yes

**Table 4:** Independent *t*-test results.

Variable	t-Value	*p*-Value	Significant Change
Digit Span Test (Memory)	4.32	<0.05	Yes
CPT (Attention)	6.25	<0.05	Yes
Stroop Test (Flexibility)	5.76	<0.05	Yes
PSS (Stress)	−5.67	<0.05	Yes
HRV (Heart Rate Variability)	4.56	<0.05	Yes

## Data Availability

The data presented in this study are available on request from the corresponding author. The data are not publicly available due to privacy restrictions.
